# Comparison of Ceramic-on-Ceramic and Ceramic-on-Highly-Crosslinked-Polyethylene in Primary Total Hip Arthroplasty: Findings of a Meta-Analysis

**DOI:** 10.7759/cureus.13304

**Published:** 2021-02-12

**Authors:** Abdulaziz Almaawi, Abduljabbar Alzuhair, Abdulaziz AlHakbani, Demah Benfaris, Fahad Al-Abdullatif, Nouf H Alabdulkarim, Waleed Awwad

**Affiliations:** 1 Orthopedic Surgery, King Saud University, Riyadh, SAU; 2 Orthopaedic Surgery, King Saud University, Riyadh, SAU; 3 College of Medicine, King Saud University, Riyadh, SAU

**Keywords:** coc, tha, hip, arthroplasty, arthritis, ceramic, polyethylene, bearing surface, cop

## Abstract

Introduction

An optimal hip implant is biocompatible, durable, and resistant to chemical and mechanical wear. This analysis aimed to compare failure (revision) and complication rates between ceramic-on-ceramic (CoC) and ceramic-on-highly-crosslinked-polyethylene (CoHXLPE) implants.

Methods

This review comprised of scientific literature published between 1995 and 2019. We included randomized controlled trials in adults (>18 years) that presented results of CoC and CoHXLPE total hip arthroplasty (THA) with more than two years of mean follow-up and drafted in English. The primary outcomes for this analysis were complications, revision rates, and loosening rates.

Results

Eight studies (1,689 hips) were included in this systematic review. There was no significant differences between COC and CoHXLPE for the risk of post-surgical complications (relative risk [RR]: 1.98, 95% confidence interval [CI]: 0.83-4.69, P = 0.12). Revision rates (RR: 1.25, 95% CI: 0.71-2.20, P = 0.43] and loosening rates between the two implants were not significantly different (RR: 1.17, 95% CI: 0.30-4.52, P = 0.82).

Conclusion

We report no significant differences between CoHXLPE and CoC in adults undergoing primary THA. Although introduced relatively recently, CoHXLPE is a cost-effective bearing that can be used for younger patients with no risk of increased complications in comparison to CoC. Further studies with longer follow-up periods are recommended to confirm the findings of this meta-analysis.

## Introduction

Demands for total hip arthroplasty (THA) continue to increase globally, as this procedure is indicated for various conditions including congenital hip disease, severe arthritis, and trauma [1,2]. It is believed that changes in population dynamics as well as the widespread use of THA in younger patients markedly account for this upsurge [2,3].

Different material couplings in THA have evolved over the last several decades to accommodate the demand, in search of an ideal prosthetic that withstands wear and tear, with the purpose of increasing implant longevity [4,5]. As a result, in recent years, several material couplings have been used while performing THA. These surface bearings include metal-on-metal (MoM), metal-on-polyethylene (MoP), ceramic-on-ceramic (CoC), ceramic-on-polyethylene (CoP), and ceramic-on-metal (CoM) [6]. Multiple factors implement in the choice of an appropriate bearing, particularly recipient’s age and level of activity. Ceramic bearings in general have proven superior to other materials for use in young, more active patients [4,7,8].

With regard to the growing literature in the topic of THA implants, identified four meta-analyses conducted by different researchers to analyse the appropriateness of a particular bearing surface. Only one of these four analyses has put the relatively recent CoHXLPE into consideration [7,9,10]. The aforementioned meta-analysis was a 2015 study that comprised RCTs comparing the three most utilized modern couplings; CoC, CoHXLPE and MoHXLPE in patients younger than 65 and found no significant difference in the risk of revision as well as the survivorship of implant across the different bearings [10]. Therefore, given the increased utilization of ceramic prosthetics, even for older patients, we performed a meta-analysis of published studies to compare the complication rates of CoC and CoHXLPE bearing surfaces in adults aged over 18 undergoing primary THA.

This meta-analysis aimed to compare (1) revision rates, (2) post-surgical complications, and (3) loosening rates between the four main types of bearing surfaces.

## Materials and methods

The search was performed using PubMed, Embase, Cochrane Library through July 11, 2019. The initial search used the subject headings ‘‘hip prosthesis’’, ‘‘arthroplasty’’, ‘‘replacement’’, and ‘‘hip’’ comparing different types of bearing surfaces using both subject headings and text words (‘‘aluminum oxide’’ or ‘‘alumina’’ or ‘‘ceramic’’; ‘‘polyethylene’’ or ‘‘polyethylene, highly crosslinked’’). Other terms used were: ‘‘implant failure’’, ‘‘prosthesis failure’’. 

We examined multiple factors that could lead to heterogeneity by using firm inclusion and exclusion criteria, following the standards proposed by the Preferred Reporting Items for Systematic Reviews and Meta-Analyses (PRISMA) statement [11]. All the studies eligible for inclusion had to be: (1) a study on adults aged >18 years; (2) a clinical study comparing the results of THA using CoC and ceramic-on-highly-crosslinked-polyethylene (CoHXLPE) with a mean follow-up of more than two years; (3) a study including any type of hip arthritis; (4) a study drafted in English. We excluded from our search (1) case reports or case series with lesser than five cases; (2) studies that reported the results of mixed cases of revision arthroplasty; (3) studies that did not present the main outcomes (number of revisions or failures). Two investigators independently reviewed the titles and abstracts of the 704 articles identified by the systematic literature search. If both reviewers agreed that a study did not meet the eligibility criteria, it was excluded. 

Data were extracted from each article and a standardized data extraction spreadsheet was used by the investigators. Data were grouped to include year, type of evidence, patient demographics, implant types, number of study participants, number of participants lost to follow-up, number of complications, implant revision events, and length of follow-up period.

This meta-analysis was performed using Review Manager 5.3 (Cochrane Collaboration, Oxford, UK). Relative risk (RR) and 95% confidence interval (CI) were calculated for dichotomous outcomes. The standardized mean difference and 95% CI are presented for continuous outcomes. Chi-square test was used for heterogeneity analysis. Heterogeneity was quantified using the I^2^ statistic. When I^2^ was ≤50%, the heterogeneity was rated as low; otherwise, it was rated as high. We used a random-effects model to calculate all effect sizes because it was more conservative and included both the random variation within the studies and the variation among the different studies. Studies performing multiple comparisons on the same treatment group or not specifying whether there was patient overlap between such repeated comparisons could result in a potential loss of independence. In such cases, adjustments were made to the weighting of studies using a described method for conservatively inflating variance estimates [12]. A funnel plot was used to assess the publication bias. Statistical significance was set at P ≤ 0.05.

## Results

**Figure 1:**
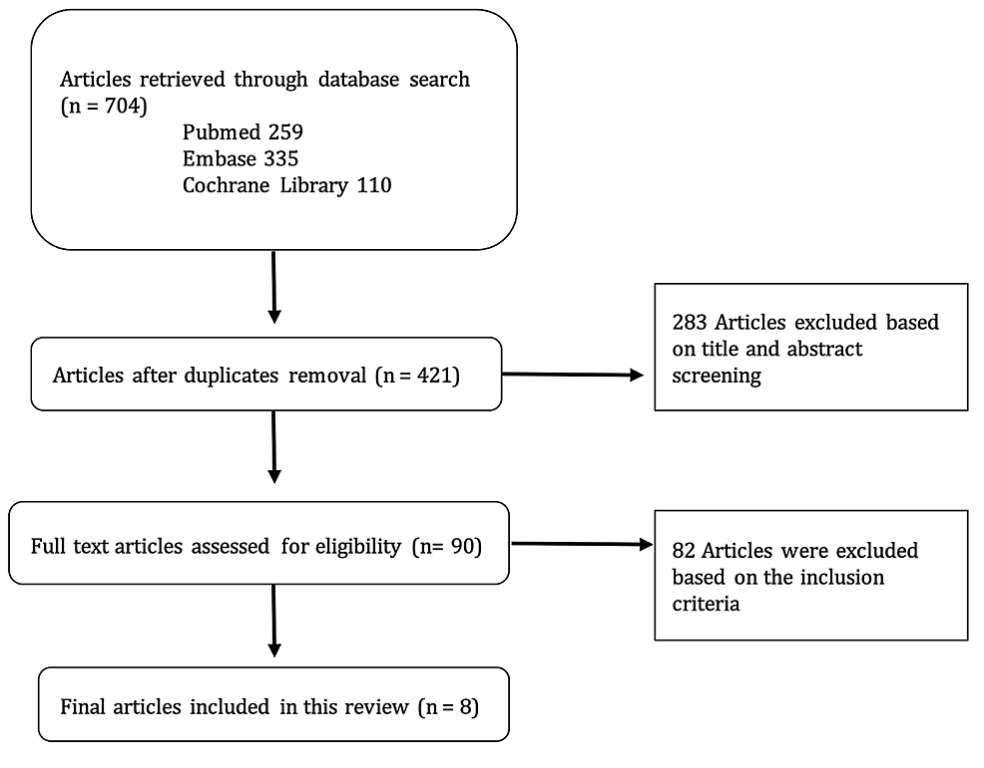
Basis of analysis.

A total of 704 identified titles and abstracts, 704 articles were judged to be relevant and were critically reviewed based on title and abstract. After duplicate removal, ninety of these articles were deemed scientifically admissible. 90 articles were independently reviewed by two investigators, 82 of which did not meet our inclusion criteria and were therefore excluded. A total of eight studies comparing CoC and CoHXLPE were considered overall, which formed the basis of this analysis (Figure 1) [7,13-19]. 

Post-surgical complication rate

**Figure 2:**
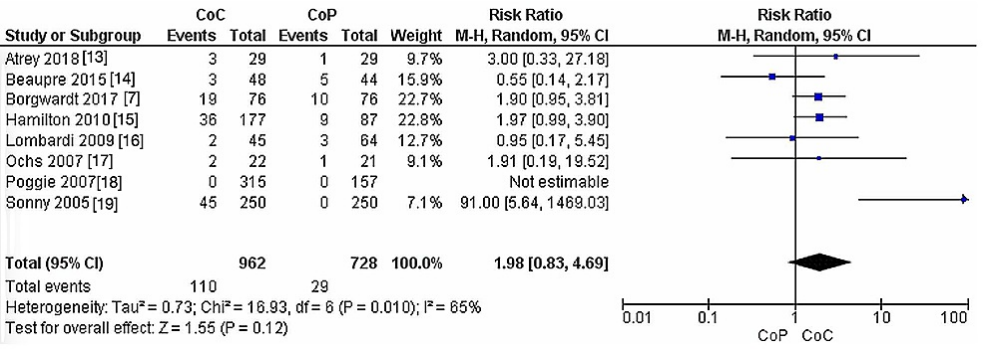
Post-surgical complication rate. CoC: ceramic-on-ceramic; CoP: ceramic-on-polyethylene; df: degrees of freedom.

In the eight studies comparing CoC to CoHXLPE in terms of post-surgical complications, no significant difference was reported between the groups (RR: 1.98, 95% CI: 0.83-4.69, P = 0.12). The heterogeneity was significant (I^2^ = 65%, P = 0.01) (Figure 2). A total of 1,689 hips were included in this analysis (Table 1).

**Table 1:** Demographic data and outcome measurement. CoC: ceramic-on-ceramic; CoP: ceramic-on-polyethylene.

	Hips in number		Mean age in years		Mean follow-up in months		Revision in number (%)	
	CoC	CoP	CoC	CoP	CoC	CoP	CoC	CoP
Atrey 2018 [13]	29	29	41.5	42.8	180	180	5 (17.2)	5 (17.2)
Beaupre 2015 [14]	48	44	53	53.6	120	120	0	3 (6.3)
Borgwardt 2017 [7]	76	76	69.1	66.4	120	120	19 (25)	10 (13.2)
Hamilton 2010 [15]	177	87	56.4	57.3	40	40	4 (2.3)	0
Lombardi 2009 [16]	45	64	75	60	73	73	3 (6.7)	3 (4.7)
Ochs 2007 [17]	22	21	56	61.5	100.8	100.8	1 (4.5)	0
Poggie 2007 [18]	315	156	54	55.3	30	30	14 (4.4)	3 (1.92)
Sonny 2005 [19]	250	250	54.97	60.9	28	28	1(0.4)	6 (2.4)

Revision rate

**Figure 3:**
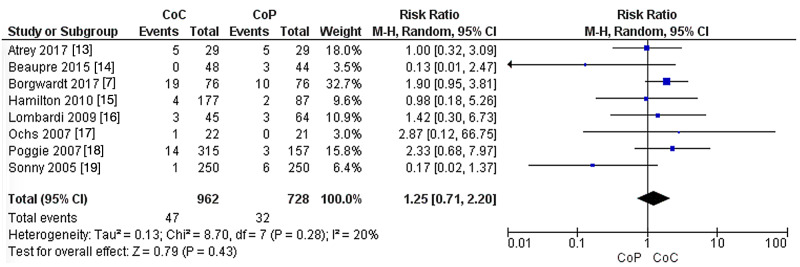
Revision rate. CoC: ceramic-on-ceramic; CoP: ceramic-on-polyethylene; df: degrees of freedom.

In the eight studies that compared CoC to CoHXLPE in terms of revision rates, no significant difference was reported between the groups (RR: 1.25, 95% CI: 0.71-2.20, P = 0.43). The heterogeneity was not significant (I^2^ = 20%, P = 0.28) (Figure 3). 

Loosening

**Figure 4:**
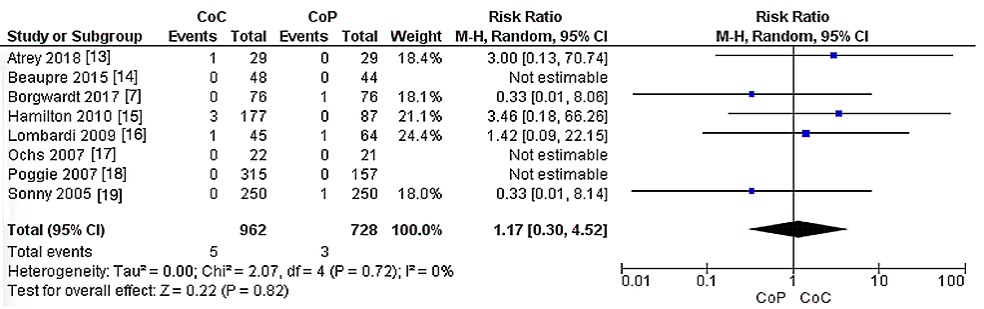
Loosening. CoC: ceramic-on-ceramic; CoP: ceramic-on-polyethylene; df: degrees of freedom.

In the eight studies that compared CoC to CoHXLPE in terms of loosening rates, no significant difference was reported between the groups (RR: 1.17, 95% CI: 0.30-4.52, P = 0.82). The heterogeneity was not significant (I^2^ = 0%, P = 0.72) (Figure 4).

## Discussion

We performed this meta-analysis to compare the post-surgical complication, revision, and loosening rates between CoC and CoHXLPE bearing surfaces in adults undergoing primary THA. This meta-analysis revealed no statistically significant difference between CoC and CoHXLPE in terms of the risk of post-surgical complications. While comparing the revision rates, we did not find any significant difference between CoC and CoHXLPE. Lastly, we also studied the loosening rates between different types of bearing surfaces and did not find any significant difference between CoC and CoHXLPE.

Arthroplasty surgeons always prefer to choose bearing surfaces with the least wear and longest survival for THA, particularly in young and healthy patients [10]. MoM, MoP, CoP, and CoC have become ubiquitous choices among arthroplasty surgeons as a substitute to conventional polyethylene, and knowing the benefits and drawbacks of each bearing surface can help in choosing the best combination for the improved long-term survival of the implant [2].

Selection of implant type depends on multiple factors, including surgeon preference, patient factors such as age and bone shape, and healthcare provider factors such as the cost of healthcare systems. Ceramic bearings were first familiarized in 1970 and became prevalent because of their lower wear rates and amplified mechanical strength [4]. However, the initial outcomes were not favourable because they resulted in higher rates of aseptic loosening and component fracture [20]. In a comparison between CoC and CoHXLPE bearing surfaces in the literature, it was found that despite differing in their implant specific complications, there was no significant difference in outcome [10]. Reported implant specific complications for CoC bearings include squeaking, fracture, and cost. Polyethylene undergoes abrasions, adhesions and fatigue, generating wear particles giving CoHXLPE bearings a higher rate of wear [4,10]. In terms of dislocation, loosening, hip function, osteolysis, and revision, the rates were comparable between the two bearing surfaces, which was reflected in the results of this study [10]. The CoC bearing surface has shown the best wear properties among all other bearing surfaces; however, it is more expensive, squeaks more, has more chances of fracture, and less modularity with fewer neck lengths [4]. Furthermore, it resulted in an increased resistance to wear and scratching. However, this has been associated with greater rates of fracture (0.004% for a third-generation CoC bearing) as opposed to other bearing surfaces [21]. Furthermore, reduced friction was observed at the bearing surface because of the smooth surface and hydrophilic properties of ceramic surfaces. This, in turn, attracts synovial fluids, resulting in reduced abrasive wear [22]. 

In regards to audible sounds (squeaking) experienced with ceramic bearings, in North America, approximately 3% of patients who underwent CoC THA experienced squeaking. Although there seems to be no valid reason for this complication, many theories have been reported in the literature. One such theory proposes that these sounds are caused by the reduced fluid lubrication between the bearing surfaces. In contrast, another theory proposed incongruity between the head and acetabular liner. A third theory presumes that the metal debris entrapped between the bearing surfaces produces sound [10,23].

However, loosening, osteolysis, and revision rates were analogous between the two bearing surfaces [23]. Given the fact that ceramic bearings are associated with reduced wear rates among all the bearing surfaces, it is considered a method of choice among young and active patients [13]. This is because it improves the longevity of the implant and lowers the risk of early revisions [22].

As factors other than the implant, including diagnosis, patient characteristics (bone quality, deformity, age, and activity levels), the skill of the surgeon, and surgical technique are regarded as extraneous factors of survival rates; long-term survival of THA has become a complex and multicausal issue. According to the Australian Orthopaedic Association National Joint Replacement Registry annual report (2018), loosening (25.6%), dislocation (21.6%), fracture (19.5%), and infection (17.7%) are considered some of the common reasons for failure and revision of conventional THA [24]. The literature, on the other hand, has shown different revision rates due to various factors such as loosening (44.1%), followed by infection (19.5%), fracture (13.6%), and dislocation (12.6%) [25].

The mean rates of revision (6% after five years and 12% after 10 years), where it is vital to examine the relationship between patient characteristics and failure modes, as well as the time of revision, to enhance THA consequences [26]. For instance, some of the prevalent reasons for revision THA are dislocation or instability, wear, loosening, and infection [27]. Studies have revealed other factors such as advanced age at primary THA, male sex, rheumatoid arthritis, and avascular necrosis as some of the main risk factors for implant failure irrespective of the implant material [25,28]. Therefore, certain factors are crucial in deciding the revisions in some patients. For example, increased operating time and male sex have been regarded as important risk factors for postoperative infections in one of the studies [29]. In contrast, advanced age, reduced head sizes, and a posterior method are associated with a higher risk of dislocation when dislocation revision is considered [28]. For instance, the findings of one meta-analysis illustrated that the fixation type is not a risk factor as assessed based on the revision rates [30].

THA is considered an extremely effective intervention, as it provides long-term functional benefits to patients. However, the need for revision is expected to incline in respect to the increase in THA demand. This meta-analysis found no significant difference between CoC and CoHXLPE in adults in terms of the risk of post-surgical complications. Revision and loosening rates between the different implants did not show any significant differences. 

## Conclusions

We report no significant differences in loosening, complication or revision rates between CoHXLPE and CoC implants used in adults undergoing primary THA. Although introduced relatively recently, CoHXLPE is a cost-effective bearing that can be used for younger patients with no risk of increased complications in comparison to CoC. Further studies with longer follow-up periods are recommended to confirm the findings of this meta-analysis.
